# The Zinc-Sensing Receptor GPR39 in Physiology and as a Pharmacological Target

**DOI:** 10.3390/ijms22083872

**Published:** 2021-04-08

**Authors:** Anna Laitakari, Lingzhi Liu, Thomas M. Frimurer, Birgitte Holst

**Affiliations:** 1 Novo Nordisk Foundation Center for Basic Metabolic Research, Faculty of Health and Medical Sciences, University of Copenhagen, Blegdamsvej 3B, 2200 Copenhagen, Denmark; anna.laitakari@sund.ku.dk (A.L.); lingzhi.liu@sund.ku.dk (L.L.); thomas.frimurer@sund.ku.dk (T.M.F.); 2 Department of Biomedical Sciences, Faculty of Health and Medical Sciences, University of Copenhagen, Blegdamsvej 3B, 2200 Copenhagen, Denmark

**Keywords:** GPR39, GPR39 agonist, zinc, zinc signaling

## Abstract

The G-protein coupled receptor GPR39 is abundantly expressed in various tissues and can be activated by changes in extracellular Zn^2+^ in physiological concentrations. Previously, genetically modified rodent models have been able to shed some light on the physiological functions of GPR39, and more recently the utilization of novel synthetic agonists has led to the unraveling of several new functions in the variety of tissues GPR39 is expressed. Indeed, GPR39 seems to be involved in many important metabolic and endocrine functions, but also to play a part in inflammation, cardiovascular diseases, saliva secretion, bone formation, male fertility, addictive and depression disorders and cancer. These new discoveries offer opportunities for the development of novel therapeutic approaches against many diseases where efficient therapeutics are still lacking. This review focuses on Zn^2+^ as an endogenous ligand as well as on the novel synthetic agonists of GPR39, placing special emphasis on the recently discovered physiological functions and discusses their pharmacological potential.

## 1. Introduction

GPR39, also known as ZnR, is a member of a large family A of 7-transmembrane (7-TM) containing G protein-coupled receptors (GPCRs) [1]. GPR39 is found in all vertebrates and was cloned together with GPR38 as structural homologues to the ghrelin receptor from human fetal brain in 1997 [2] and belongs to the ghrelin receptor subfamily with six of its most closely related receptors [3]. The other members of this family are well-established regulators of metabolism through their ligands: the peptide hormones and neuropeptide regulators ghrelin, motilin, neurotensin and neuromedin U [3]. Conversely, no endogenous peptide ligand has been discovered for GPR39 [4]. Coincidentally, the existence of an ionic zinc (Zn^2+^) -sensing receptor was indicated in 2001, when extracellular Zn^2+^ was found to activate intracellular Ca^2+^ signaling; the receptor then named the Zn^2+^-sensing receptor (ZnR) and now known to be GPR39 [5]. Indeed, physiological concentrations of Zn^2+^ were shortly after shown to activate GPR39 [6,7,8].

Zinc is essential for human health and zinc deficiency is recognized as a world-wide malnutrition problem, affecting 17% of the global population [9]. All cell types require Zn^2+^ for various catalytic and regulatory functions and around 3000 proteins are estimated to have binding sites for Zn^2+^ [9,10]. Consequently, zinc deficiency affects the functions of multiple tissues and cell signaling pathways; for example, zinc plays a role in immune responses, DNA replication and repair, oxidative stress response, aging, cell cycle progression, homeostasis and apoptosis [9,11,12,13]. Extracellular Zn^2+^ concentration can increase in many circumstances, such as when Zn^2+^ is co-released from vesicles with insulin in the pancreatic β-cells or with glutamate in the nerve synapses, or in connection with cell death and injury, which then triggers GPR39-dependent signaling [14,15,16]. This review aims to reveal the therapeutic potential of GPR39 activation by Zn^2+^ supplementation in the absence and presence of the novel synthetic GPR39 agonists, characterizing them and placing special emphasis on the newly discovered physiological functions of GPR39.

## 2. GPR39 Structure and Signaling

The gene encoding for GPR39 is located in the chromosome 2, locus q21.2, and is around 230 kb in size. The gene encoding the full-length 435 amino acid long (52 kD) GPR39-1 constitutes two exons, separated by an intron. The first exon consists of 285 amino acids and contains the transmembrane (TM) domains I–V and the second, consisting of 150 amino acids, the TM domains VI and VII. [2]. A truncated, 32 kD isoform containing the first five TM domains, GPR39-1b, has also been identified. This splice variant is likely to be inactive and does not dimerize with the full-length GPR39-1a, but has been shown to function as a dimerization partner to neurotensin receptor 1 (NTSR1), thus having a role as a negative regulator of NTSR1 signaling [17,18].

Upon ligand binding, GPCRs undergo conformational changes resulting in intracellular G-protein activation and subsequent downstream signaling [19]. GPR39 activation induces signaling pathways that regulate various cellular functions, such as survival, proliferation, differentiation, and ion transport [5,20,21,22] and has been shown to signal through Gα_q_, Gα_s_, Gα_11/12_ and β-arrestin recruitment [6,8,23,24]. When the downstream signaling upon GPR39 activation is mediated by Gα_q_, phospholipase C (PLCβ) is activated, resulting in inositol 1,4,5-triphosphate (InsP_3_) generation, which in turn stimulates Ca^2+^ release from its endoplasmic reticulum (ER) storages. The intracellular Ca^2+^ results in extracellular signal-regulated kinase (ERK1/2) and subsequent ERK/mitogen-activated protein kinase (MAPK) signaling pathway activation, and GPR39 activation also increases protein kinase B (AKT) phosphorylation and AKT/phosphoinositide 3-kinase (PI3K) signaling pathway activation [5,8,25,26,27]. These two pathways contribute to cell survival and proliferation, respectively [28,29]. Moderate cAMP response element (CRE)-mediated transcription induction is observed with GPR39 [6,8], mainly mediated by Gα_s_-induced cAMP production and protein kinase A (PKA) signaling [6]. GPR39 signaling is also able to induce Gα_12/13_ activation, which via PI3K and Ras homolog family member A (RhoA) induces serum response element (SRE)-mediated transcription [6,8]. GPR39 also triggers arrestin transport to the plasma membrane [6]. Recently, several studies have indicated GPR39 to interact with the NF-κB signaling pathway. However, all of these studies achieved their results by using a synthetic GPR39 agonist; thus, the question remains whether Zn^2+^ is able to activate this pathway in physiological conditions [30,31,32].

GPR39 has relatively high ligand-independent constitutive activity, based on an aromatic cluster on the inner face of the extracellular ends of TM domains VI and VII, similarly to other members of the ghrelin receptor family [8,33]. Gα_q_ is the mediator of this, activating PLCβ, which results in an induction of InsP_3_ turnover. Activation of the ERK/MAPK pathway is not involved in the constitutive activity, which thus results only in modest CRE-mediated transcription induction. Constitutive GPR39 signaling was also able to induce Gα_12/13_ activation, and via PI3K and RhoA inducing robust serum response element (SRE)-mediated transcription [6,8]. The constitutive activity did not induce cAMP production, suggesting that Gα_s_ is not involved [6]. However, a recent report suggested that the constitutive signaling of GPR39 varies between vertebrates. Whereas with human, chicken and frog the phylogenetic and selection analysis indicated Gα_q_ and Gα_11/12_, zebrafish were shown to additionally constitutively activate Gα_s_ [34]. The physiological roles of the constitutive activity are not clear, but it is important to note that due to it, variations in the expression of GPR39 also directly affect the potency of its downstream signaling.

A negative feedback method desensitizing GPR39 for further activation has been shown to take place and is important due to Zn^2+^ not being degraded like most ligands of GPCRs. A robust desensitization of GPR39 follows exposure to high concentrations of Zn^2+^, which can result in complete loss of GPR39 signaling dose- and time-dependently [14,24,35,36]. Internalization of membrane receptors into vesicles is a common method of desensitization for the GPCRs [37]. GPR39 was reported not to undergo basal internalization [8,38] and ZnCl_2_-induced internalization occurred at a significantly lower level compared to the ghrelin receptor in GPR39-overexpressing HEK293 cells [38]. However, GFP-tagged GPR39 was shown to internalize when stimulated simultaneously with Zn^2+^ and a GPR39 agonist TC-G 1008 [35]. The desensitization and internalization of GPR39 were shown to occur via Gα_12/13_ and RhoA [35], instead of internalization by β-arrestin, more typical to GPCRs [37].

GPR39 has been shown to be highly phosphorylated in basal conditions; the level of phosphorylation not further increased by Zn^2+^ [38]. Which kinase is responsible for this is still unknown, although one study suggested that G protein-coupled receptor kinase 2 (GRK2) might be involved [35].

GPCRs are known to form heteromeric complexes with other G proteins [39]. When GPR39 was co-expressed with the GPCRs 5-hydroxytriptamine 1A receptor (5-HT_1A_) and galanin receptor 1 (GalR_1_), GPR39 formed heterodimers with 5-HT_1A_ and heterotrimers with both receptors in HEK-293S GnTi^−^ cells [40]. The GPR39-5-HT_1A_ complex was shown to increase SRE induction compared to GPR39 alone, suggesting that 5-HT_1A_ might enhance GPR39 activity, but the presence of GalR1 blocked GPR39 signaling. Zn^2+^ was shown to modulate the interaction of GPR39 with the other receptors and to regulate the balance between receptor complex formation; at low Zn^2+^ concentrations the dominant complex was 5-HT_1A_-GalR_1_ and GPR39 was absent, whereas at higher Zn^2+^ concentrations, complexes with GPR39 were dominant [40]. Another extracellular cation sensing GPCR, the Ca^2+^ sensing receptor (CaSR), was suggested to interact and regulate GPR39 functionally when co-expressed in prostate cancer (PC3) or ductal salivary gland (HSY) cells [27]. GPR39 was shown to directly interact with CaSR by co-immunoprecipitation, and CaSR activity was shown to promote GPR39 surface expression and signaling, as treatment with a CaSR agonist increased the GPR39-dependent Ca^2+^ response [27]. Additionally, GPR39 has been suggested to interact with a calcium-activated anion channel, transmembrane member 16A (TMEM16A) [41]. GPR39 activation in primary intestinal fibroblast-like cells resulted in TMEM16A-dependent currents and membrane depolarization, suggesting that GPR39 activation is functionally linked to TMEM16A channel opening [41]. Additionally, the cAMP-dependent protein kinase A inhibitor β (PKIB) was identified as an interacting partner for GPR39 in a yeast-2-hybrid screen [42]. When co-expressed in Chinese hamster ovary cells, GPR39 co-localized with PKIB, resulting in an increase in its constitutive activity, but PKIB had no effect on the Zn^2+^-dependent activation of GPR39. Contrariwise, Zn^2+^ treatment resulted in a dissociation of PKIB from GPR39, switching the signaling from constitutive to ligand-dependent [42]. This suggests that Zn^2+^ is not only an agonist of GPR39, but also an important regulator of constitutive vs. ligand-dependent GPR39 signaling. In C2C12 myoblast cells, Pax7 has been shown to activate the transcription factor Zac1 in order to regulate GPR39 expression. GPR39 was suggested to be a direct target gene of Zac1 [43]. GPCR dimerization has been widely shown to affect their activity and affinity [39], which these studies suggest to be also accurate for GPR39. However, it is yet to be confirmed whether GPR39 oligomerization or the other interactions discussed here happen in physiological conditions, since most studies to date are performed in overexpression models in heterologous expression systems.

## 3. Ligands of GPR39

Initially, obestatin, a peptide encoded by the ghrelin gene, was suggested to be an endogenous ligand for GPR39, reducing food intake and gastric motility [23]. However, it was later confirmed that obestatin does not activate GPR39 [4,6].

### 3.1. Endogenous Ligands of GPR39: Zn^2+^

Zinc was first indicated to activate GPR39 in 2004 [8], and in 2007 two research groups showed that GPR39 senses changes in extracellular zinc concentrations, which results in intracellular signaling pathway activation [6,7]; although the existence of a Zn^2+^-sensing receptor was already suggested in 2001 by functional identification [5]. Zn^2+^ is found in all tissues in relatively high concentrations [9] and has been so far shown to activate GPR39 in bones, neurons, pancreatic cells, salivary gland cells, epithelial cells of the colon (colonocytes), epidermal cells of the skin (keratinocytes), skin fibroblasts, endothelial cells, cardiac valve interstitial cells, aortic vascular smooth muscle cells and prostate cancer cells [14,24,26,36,44,45,46,47,48,49,50]. Extracellular Zn^2+^, able to activate GPR39, can be released from the pancreatic β-cells together with insulin, nerve synapses together with glutamate, salivary gland vesicles, Paneth cells of the intestinal crypts or in connection with cell injury and death [14,15,16,51,52].

Importantly, endogenous physiological Zn^2+^ concentrations following release from vesicles or injury are able to activate GPR39 [14,15,21,47,53]. Physiological Zn^2+^ concentrations are in the nano-micromolar range, varying between tissues [9]. For example, in colonocytes the EC_50_ for GPR39 is 80 µM [26] whereas in keratinocytes it is in the nanomolar range [14], corresponding to the higher available Zn^2+^ concentrations in the colon compared to the skin [9]. Zn^2+^ binding to GPR39 and its consequent activation works concentration-dependently, resulting in the activation of all GPR39 mediated pathways, and signaling through Gα_q_, Gα_s_, Gα_11/12_ and β-arrestin recruitment [6,8,23,24]. The Zn^2+^ binding sites in GPR39 have been identified as histidine residues His17, His19 and aspartate residue Asp313 [54], and are conserved in the receptor of all vertebrates, excluding fish [55]. These residues have been shown to be pH sensitive; hence, physiological changes in extracellular pH directly affect and regulate GPR39 signaling [56]. pH levels both below and above the physiological 7.4 reduce GPR39 signaling drastically, eliminating the intracellular Ca^2+^ response and phosphorylation of PI3K completely at pH 6.4 [57], suggesting that GPR39 is strongly optimized for neutral pH levels. Fish GPR39 does not seem likely to be able to bind Zn^2+^; since GPR39 is present in fish, this could be an indication of another endogenous ligand existing for GPR39 in fish and possibly in other vertebrates [58]. The Zn^2+^-dependent activation of GPR39 has been characterized in many physiological and pathological conditions, reviewed in detail in Section 4. Ionic zinc is so far the only identified endogenous ligand for GPR39. However, it cannot be excluded that another endogenous ligand exists, since Zn^2+^ also has various other interaction partners [9,10], and novel synthetic ligands have been shown to be able to increase GPR39 signaling further than achieved solely by Zn^2+^, as will be discussed below. It is thus also possible that zinc is simply an enhancer and coactivator of another endogenous ligand, yet to be identified.

### 3.2. Synthetic Ligands of GPR39

Only a small number of synthetic ligands for GPR39 have been characterized. The best characterized and most widely published agonist to date is TC-G 1008 (originally known as GPR39-C3), but since its discovery, other potent agonists have been characterized. Since the first GPR39 agonist was discovered in 2013 [59] and shortly after in 2014 TC-G 1008, which quickly became commercially available [60], the number of studies focusing on GPR39 agonists has been steadily increasing in recent years. This has permitted the characterization of the physiological functions of GPR39 more specifically, compared to the GPR39-deficient or overexpressing mice, where global systemic compensatory mechanisms may play a role.

The first GPR39 agonist was a piperazine derivative, identified by a screening of GPCR-focused libraries against GPR39 [59]. The agonist was able to activate GPR39 and to induce the intracellular Ca^2+^ response, but turned out to be only moderately potent and not suitable for in vivo studies [59]. Soon after, in 2014, another research group identified 2-pyridylpyrimidines as potential GPR39 agonists via a reporter gene assay designed for identifying inhibitors of Hedgehog signaling, and then optimized them to improve efficiency and pharmacokinetics, which yielded TC-G 1008 [60]. It is a highly potent and selective GPR39 agonist with EC_50_ values of <1 nM, and orally bioavailable. When TC-G 1008 treatment was combined with Zn^2+^, more InsP was generated than with either substance alone, indicating that TC-G 1008 does not compete for binding with Zn^2+^, but the presence of Zn^2+^ further potentiates GPR39 activation by TC-G 1008. Originally, TC-G 1008 was also found to robustly induce glucagon-like peptide-1 (GLP-1) levels when orally administered to mice [60]. Later, it has been shown to activate all GPR39 inducible pathways [35]. Since its discovery, TC-G 1008 has proven to be a potent agonist and a very valuable tool in characterizing the physiological functions and therapeutic potential of GPR39 activation, published by a number of studies to date [30,31,32,35,61,62,63,64,65,66,67,68,69,70,71,72]. However, most studies have not verified their findings in GPR39^−/−^ settings, which would be critical in order to confirm that the observed effects are indeed GPR39-dependent.

Additionally, in 2014, a series of cyclohexyl-methyl aminopyrimidine chemotype compounds (CMAPs) activating GPR39 was identified co-incidentally, while similarly screening for compounds that could inhibit Hedgehog signaling [73]. The identified compounds, which inhibited Hedgehog signaling, were also found to increase InsP production, indicating GPCR involvement. GPCR mRNA expression levels were correlated with the compound activity in different cell lines, which indicated the target of the compound to be GPR39, later verified with GPR39-deficient cells. The EC_50_ of the most potent compound CMAP 7 was 20 nM, thus the compound was not as potent as TC-G 1008. Importantly, this study showed that GPR39 activation interferes with Hedgehog signaling, which plays important roles in tumorigenesis and determines cell fate during development [73]. Shortly after, in 2015, three novel GPR39 agonists were described, AZ7914, AZ4237 and AZ1395 [74]. They were identified by a high throughput screen of the AstraZeneca compound library and were verified by a limited medicinal chemistry program. These compounds were also found to be more potent in the presence of Zn^2+^ and to interact directly with Zn^2+^. In a physiological characterization, these agonists were not able to improve glucose tolerance in lean or obese mice or in Zucker fatty rats. All three compounds had higher EC_50_ values than TC-G 1008, thus being less potent [74].

In 2016, two previously existing kinase inhibitors, the Janus kinase (JAK) inhibitor LY2784544 and the PI3Kβ inhibitor GSK2636771, were identified as novel GPR39 agonists by unbiased small-molecule-based screening of multiple compound libraries using a modified β-arrestin recruitment assay [65]. Their signaling patterns were compared to those of TC-G 1008, and GPR39 activation by all three compounds was shown to be allosterically modulated by physiological concentrations of Zn^2+^. As with TC-G 1008, the presence of Zn^2+^ increased the potency of LY2784544 and GSK2636771 to activate GPR39 and downstream signaling. Although LY2784544 and GSK2636771 were suggested to be more potent in activating GPR39 than inhibiting JAK2 and PI3Kβ, respectively, to date, these compounds have not been utilized in other GPR39 studies, likely due to not being exclusively selective for GPR39 [65]. However, one study used LY2784544 as a control for TC-G 1008 and found that both compounds similarly suppressed lipopolysaccharide (LPS) –induced interleukin 6 (IL6) production in macrophages [62]. Furthermore, as both compounds are being tested in clinical trials, their potential to activate GPR39 would be of great interest to study, as GPR39 activation might induce adverse effects or be partly responsible for the positive outcomes. Currently, according to www.clinicaltrials.gov, LY2784544 is being tested in myeloproliferative disorders and GSK2636771 in various cancers, most focusing on cancers with genetic changes of phosphatase and tensin homolog (PTEN).

Another novel set of GPR39 agonists were discovered in 2017 with the help of a homology model-based approach [75]. This model took advantage of a suggested binding site for synthetic ligands in GPR39 and commercially available libraries of synthetic compounds. Interestingly, some of the most potent identified compounds turned out to be inactive alone, but highly potent and selective in the presence of Zn^2+^ as an allosteric enhancer. Mutational mapping indicated that the synthetic agonists are able to bind to the main ligand binding pocket of GPR39, whereas Zn^2+^ seems to bind differently when acting as the sole agonist as compared to acting as an allosteric modulator. The selected agonist, TM-N1324 (compound 8 in [75]), had an EC_50_ of 280 nM and 9 nM in the absence and presence of Zn^2+^, respectively, thus being potent even in the absence of Zn^2+^. TM-N1324 could be orally administered and resulted in micromolar plasma concentrations, adequate for maximal activation of GPR39. With the help of this agonist, GPR39 was identified as an important regulator of gastric somatostatin secretion, increasing somatostatin secretion and correspondingly decreasing ghrelin secretion in primary gastric mucosal cells; an effect which was eliminated in GPR39^−/−^ cells [75,76].

The first biased ligand, i.e., a ligand favoring selected signaling pathways over others, for GPR39 was discovered in 2017 [35]. GSB-118 was found through high-throughput screening of a compound library and by utilizing cAMP assays in the presence of Zn^2+^. It showed functional selectivity by activating the Gα_s_ pathway, resulting in cAMP responses and β-arrestin recruitment, but did not activate the Gα_q_ or Gα_12/13_ pathways, result in InsP accumulation or Ca^2+^ response, or desensitize GPR39. GSB-118 did not show any independent agonist activity but acted as a positive allosteric modulator for Zn^2+^, potentiating its responses [35]. The lack of desensitization observed here could attenuate drug tolerance and therefore, enhance responses to treatment. Development of more biased agonists targeting certain pathways more specifically rather than activating all GPR39-dependent signaling would allow for more targeted therapies and might be a key for future applications of GPR39 agonism for therapeutic purposes. More knowledge about the physiological functions of GPR39 and their molecular mechanisms in each disease is needed, before the development of signaling pathway-targeted therapies is meaningful. However, all identified synthetic ligands have not been fully characterized regarding signal transduction and might include some biased agonists. Despite being highly potent, the majority of these novel agonists presented here are more or less Zn^2+^-dependent, and thus it remains to be seen if they are sufficiently functional in tissues with lower physiological Zn^2+^ concentrations, and importantly, if they have therapeutic potential in conditions linked to Zn^2+^ deficiency. It is also still an open question whether at least some of the synthetic ligands simply function as allosteric activators of Zn^2+^ and not vice versa, as was suggested for GSB-118 [35]. Additionally, the GPR39-specificity of most ligands in physiological conditions will still need to be determined in GPR39^−/−^ cell and tissue models.

## 4. Physiological Functions of GPR39

**Figure 1:**
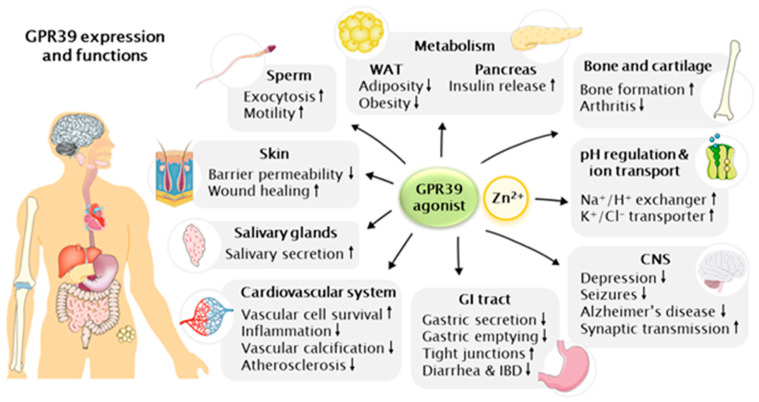
A schematic illustrating GPR39 expression and functions in different tissues. Abbreviations: CNS, central nervous system; GI, gastrointestinal; IBD, inflammatory bowel disease; WAT, white adipose tissue. This figure was created using images from Servier Medical Art (http://smart.servier.com, accessed on 5 March 2021), licensed under a Creative Commons Attribution 3.0 Unported License.

The most abundant GPR39 expression is found in the endocrine and metabolic tissues; liver, pancreas, gastrointestinal (GI) tract and kidney, and lower levels in adipose tissue, heart, brain, skin, cartilage, bone and thyroid [2,6,14,17,23,45,46,72,77,78,79,80,81,82,83,84,85]. The absence of a known endogenous ligand for GPR39 has also kept its physiological roles relatively unidentified. Knockout and overexpression rodent models have been able to shed light on them and more recently the synthetic ligands for GPR39 have been used for unraveling many new functions: indeed, GPR39 seems to be involved in many important metabolic and endocrine functions in the variety of tissues it is expressed, as presented in Figure 1 and reviewed in more detail here.

### 4.1. pH Regulation and Ion Transport

GPR39 has been shown to regulate ion transport: its activation induces the activity of the Na^+^/H^+^ exchanger (NHE) in neurons, keratinocytes and colonocytes [14,26,57], where ion transport plays essential roles in physiology and intracellular pH homeostasis [86]. During neuronal activation, H^+^ accumulates inside neurons, affecting their excitability [87]. Zn^2+^-induced GPR39 activation increases NHE activity, supporting the restoration of pH homeostasis in the neurons [57]. Similarly, colonocytes face pH challenges continuously, as non-digestible carbohydrates decrease the fecal pH and protein increases it [88], and GPR39 activation was shown to restore their pH by inducing NHE activity [26]. The activation of NHE via GPR39 signaling lowers the extracellular pH by H^+^ export, and in keratinocytes this has been suggested to be required for the establishment of a permeability barrier [89]. It is not yet clear which proteins mediate the GPR39-induced NHE activation, though Gα_q_ coupling has been shown to affect it in other receptor families [90].

GPR39 signaling also activates K^+^/Cl^−^ co-transporters KCC1 and KCC2. KCC1 is widely expressed and plays a role in reducing cell swelling by mediating Cl^−^ efflux [91]. KCC2 is expressed in brain neurons and is responsible for maintaining a low intracellular Cl^−^ concentration required for the fast synaptic inhibition via GABA and glycine [92,93]. GPR39 activation participates in the essential KCC2 mediated Cl^−^ transport by increasing KCC2 expression and Cl^−^ efflux [47]. KCC1 was shown to be activated in colonocytes GPR39 dependently, mediated by MAPK, and resulting in an upregulation of Cl^−^ transport [94]. Due to KCC1 being broadly expressed, various effects mediated by GPR39 activation are likely to be still discovered in different tissues. KCC2′s possible but yet unknown involvement in neuronal diseases would open therapeutic possibilities via GPR39 activation. Very recently, two studies have indicated that GPR39 also activates KCC3, a modulator of cell volume, in breast cancer cells [95] and in cellular protrusions, which are essential for cell migration [96]. The GPR39-dependent activation of KCC3 in cellular protrusions lead to actin re-organization, essential for the formation of the protrusions and induction of matrix metalloproteinase (MMP) release, crucial in extracellular matrix (ECM) degradation and cell movement. Thus, this study indicates the role of GPR39 in regulation of cell proliferation, migration and invasion via KCC3 activation [96].

### 4.2. Neuronal Functions

Zinc is indicated to have a role in many neuronal disorders, as reviewed in [97]. Multiple regions of the brain contain a subclass of Zn^2+^ containing glutamatergic neurons [16] where the storage of Zn^2+^ in the synaptic vesicles is facilitated by its transporter, zinc transporter 3 (ZnT3) [98]. When the synapses are activated, Zn^2+^ is released together with glutamate [99,100]. GPR39 is expressed in the brain in the hippocampus and amygdala [82] and Zn^2+^ released from synaptic vesicles has been shown to activate GPR39 [101]. Hippocampal tissue slices were stimulated with electricity to induce Zn^2+^ release, which resulted in increased intracellular Ca^2+^ levels. The response was shown as Zn^2+^-dependent due to calcium disodium ethylene diamine *tetra*-acetate (CaEDTA) chelation of Zn^2+^ inhibiting it. Additionally, the response persisted but was much weaker in the hippocampi of mice deficient of ZnT3 and thus, Zn^2+^ in the synaptic vesicles is important for the response [101]. Furthermore, ZnT3^−/−^ mice are reported to be predisposed to seizures and having defects in neural development and cognitive behavior [102,103,104].

Zinc deficiency is often diagnosed in connection with depression and zinc supplementation has been shown to reduce its symptoms [105]. Changes in GPR39 expression have been reported in depression, showing a correlation with depression-related behavioral changes and suicide [106,107]. The role of GPR39 in depression was also indicated by the inhibition of serotonergic or noradrenergic and dopaminergic transmission, which led to GPR39 downregulation in mice [108]. GPR39 was upregulated in the frontal cortex following chronic (but not acute) antidepressant treatment of zinc-deficient mice [109] and GPR39^−/−^ mice showed depressive-like behavior and anxiety-like phenotypes in behavioral tests, which was connected to a downregulation of cAMP response element-binding protein (CREB) and brain-derived neurotrophic factor (BDNF) in their hippocampi [110]. BDNF is an important neurotrophic factor for synaptic plasticity and neuronal survival, and its expression in patients with depression is decreased [111]. Correspondingly, treatment of GPR39-deficient hippocampal cells (HT-22) with corticosterone resulted in decreased expression levels of the anti-apoptotic CREB, BDNF and BCL-2 mRNAs and increased levels of pro-apoptotic proteins, whereas wild-type (WT) cells simultaneously treated with the GPR39 agonist TC-G 1008 had the opposite expression levels [67]. The GPR39-induced anti-apoptotic mRNAs correlated with cell viability, apoptosis and mitochondrial membrane potential, suggesting GPR39 to be neuroprotective [67]. Recently, combining the traditional fluoxetine anti-depression drug with zinc hydroaspartate for the treatment of rats on a chronic mild stress model resulted in higher hippocampal levels of antioxidant enzymes, metallothioneins, GRP39 and BDNF than treatment with either of the two compounds alone [112]. Metallothioneins are important for Zn^2+^ homeostasis and were induced by fluoxetine, whereas BDNF induction was shown to be GPR39-dependent, and more robust due to the fluoxetine-induced metallothionein involvement [112]. Additionally, a potential explanation for the connection between GPR39 and depression was suggested to be based on the heteromeric complex formation of GPR39 with 5HT_1A_ and GalR_1_, two receptors whose role in major depressive disorder has been confirmed [113]. The study reported that from the possible dimeric and trimeric GPR39-5HT_1A_-GalR_1_ complexes, the pro-depressive receptor complexes not including GPR39 were dominant at low Zn^2+^ concentrations, whereas at higher Zn^2+^ concentrations GPR39 signaling increased in complex with 5HT_1A_, which is suggested to act as an anti-depressive complex [40]. However, when the antidepressant response of TC-G 1008 on mice was studied by behavioral challenges, the results were inconsistent. TC-G 1008 induced an antidepressant response in the forced swim test, but inconsistent results in the light/dark test and no difference in the elevated plus maze test, likely explained by an observed sedative effect of TC-G 1008. However, TC-G 1008 upregulated GPR39 and BDNF expression in the hippocampi [70]. Another study reported that the antidepressant effect of TC-G 1008 lasted up to 24 h after a single dose, longer than the effect of two established antidepressant drugs, imipramine and MK-801 [69]. Chronic treatment with TC-G 1008 resulted in a similarly decreased immobility time in the forced swim test as with the established antidepressant drugs, and a slight upregulation of BDNF was observed [69]. Whether zinc deficiency and changes in GPR39 expression play a causal role in the pathogenesis of depression is still unknown. The therapeutic potential of GPR39 agonism in the treatment of depression seems promising but requires further studies. However, combining GPR39 activation with the traditional depression medications seems to be a valid alternative, and would be of importance to be studied with the synthetic agonists instead of Zn^2+^, which could further improve the outcomes.

Zinc deficiency has been indicated by various studies in connection with an increased risk for seizures [114,115], studied also extensively in children [116,117]. Knockout of the zinc receptor ZnT3 results in a lack of zinc in the synaptic vesicles and consequently has been shown to result in seizures and neuronal damage [97]. A possible role for GPR39 in epilepsy was revealed when a deficiency of KCC2 was shown to result in an increased risk of seizures due to impaired intracellular Cl^−^ regulation [118,119]: the susceptibility to kainic acid-induced seizures was shown to be drastically increased in GPR39^−/−^ mice, having more frequent and more severe seizures compared to WT. This was shown to be KCC2 mediated via Gα_q_ and ERK1/2 signaling and inhibited by Zn^2+^ chelation. This study indicated that the increased KCC2 activity by GPR39 activation during seizures could help restore homeostasis and thus, GPR39 could be targeted pharmacologically to decrease the severity and duration of epileptic seizures [120]. Feeding a Zn^2+^-deficient diet to WT rats in a model of lithium chloride-pilocarpine-induced developmental seizures resulted in an exacerbation of long-term seizure effects and a loss of GPR39 expression in the hippocampi. Zn^2+^ supplementation improved the seizure effects and upregulated GPR39 and myelin basic protein. The study indicates a possible role for GPR39 in seizures but did not characterize whether the effects were GPR39-dependent [121]. Zn^2+^-induced KCC2 activation is required for restoring ion homeostasis and decreasing neuronal activation, which plays an even more important role when neuronal excitability is increased in conditions such as epilepsy. Zinc or GPR39 deficiency can prevent this mechanism, resulting in worse seizures [122].

The dyshomeostasis of Zn^2+^ in the brain has been extensively studied and shown to play an important role in the pathogenesis of Alzheimer’s disease (AD) [123]. AD is characterized by an accumulation of amyloid β, which has been shown to bind to Zn^2+^, reducing its availability [124,125]. The GPR39-induced Ca^2+^ response and the following ERK phosphorylation in neurons pre-treated with amyloid β were shown to be hindered [126]. Selenium (Se) has been indicated to be protective against AD due to its antioxidant properties [127]. Combined treatment with Zn^2+^ and Se of rats with streptozotocin-induced AD indicated better protection against reactive oxygen species, mitochondrial membrane damage and cognitive decline than treatment with Zn^2+^ or Se alone. Interestingly, GPR39 mRNA expression was reported to be downregulated in all AD groups compared to the control group and was unaffected by Zn^2+^ treatment [128]. Further research is required to confirm whether these effects were mediated by GPR39.

### 4.3. Skin and Wound Healing

Zinc deficiency resulting in reduced wound healing is well established, and correspondingly, the application of zinc creams enhances the wound healing process, as reviewed in [129]. Skin, as well as other proliferating tissues, contains a lot of zinc, suggesting a physiologically important role for it, and zinc transporters have been shown to have an essential role in skin formation [130]. GPR39 was shown to be activated by Zn^2+^ released from human keratinocytes (HaCaT) upon injury, and treatment of the keratinocytes with Zn^2+^ induced a Ca^2+^ response, which was eliminated when GPR39 expression was inhibited [14]. Similar results were obtained in another study using primary keratinocytes [5]. A scratch closure assay demonstrated that cell proliferation was induced by Zn^2+^, presumably via the GPR39-dependent activation of the MAPK pathway and a following NHE-1 activation, which is essential for cell migration and polarization but also, for decreasing barrier permeability. The response was inhibited by a PLC inhibitor or chelation of Zn^2+^, indicating Zn^2+^-induced GPR39 activation to be in a key role in epithelial repair initiation [14]. A recent study demonstrated that GPR39 activation by TC-G 1008 in HaCaT cells increased proliferation and ERK phosphorylation concentration-dependently, which co-treatment with PI3K and mitogen-activated protein kinase kinase (MKK) inhibitors suppressed [64]. Additionally, a recent study reported that GPR39 plays a role in mast cell-induced wound healing [48]. When activated, mast cells released Zn^2+^ via ZnT2, triggering skin fibroblasts to produce the cytokines IL-6 and tumor necrosis factor α (TNFα) GPR39-dependently, which promoted wound healing. Mouse embryonic fibroblasts (MEFs) with GPR39 knockdown had markedly reduced cytokine expression, and wound healing was impaired in mice deficient of GPR39 or IL-6. The effects were shown to be mediated by GPR39 via the MAPK/ERK pathway [48]. Hence, GPR39 signaling seems to be responsible for the Zn^2+^-induced wound healing, and these results suggest potential for GPR39 agonists to be used to promote wound healing and induce keratinocyte proliferation. According to these studies, GPR39 seems to also play a role in inflammation, which could be of significance in a variety of tissues and conditions, and due to the observed effects on barrier permeability, GPR39-induced NHE-1 activation could also be involved in preventing an intrusion of harmful bacteria and thus, infection.

### 4.4. Metabolic Diseases

The studies of GPR39 in satiety control and obesity were initially motivated by obestatin being suggested as an endogenous ligand of GPR39, as well as due to a few other members of the ghrelin receptor family taking part in satiety regulation. However, despite obestatin not being involved, Zn^2+^ has been indicated to play a role in obesity and adipocyte biogenesis [131], and GPR39 is expressed in adipose tissue at a relatively high level [17], indicating a physiological importance there. One study reported no differences in body weight or adiposity between 24-week-old GPR39^−/−^ and WT mice fed chow diet [132], whereas another study showed that GPR39^−/−^ mice fed chow diet had higher body weight, more adipose tissue and higher plasma cholesterol levels than WT mice, but differences were only observed after the age of 16–17 weeks and became more prominent as the mice aged up to 85-weeks-old [133]. In agreement, another study showed that following a high-fat diet (HFD), GRP39-deficient mice had gained significantly more weight than WT and had an increased fat mass due to a deficiency in increasing thermogenesis during the HFD. Altered adipocyte metabolism was observed in the GPR39-deficient mice, who had decreased levels of hormone-sensitive lipase (HSL) and adipose triglyceride lipase (ATGL), resulting from the ERK1/2 pathway being downregulated [134]. All three studies reported no difference in food intake between GPR39^−/−^ and WT mice. GPR39 has also been indicated to have a role in the proliferation and differentiation of intramuscular preadipocytes via the activation of the PI3K/AKT pathway, whose inhibition blocked the effect [22]. The overexpression of GPR39 in preadipocytes resulted in an increased expression of the adipogenic genes, peroxisome proliferator-activated receptor gamma (PPARγ), CCAAT/enhancer-binding protein α (C/EBPα) and adducin-1 (ADD1), and downregulated pro-apoptotic caspase-9 [22].

In the pancreas, GPR39 localizes in the pancreatic islet β-cells and ductal cells lining the pancreatic ducts [45]. Insulin-containing secretory vesicles contain Zn^2+^ due to abundant ZnT8 zinc transporter activity [135,136]. Zn^2+^ is released from the vesicles with insulin [15], which suggests that it might activate GPR39 signaling in an autocrine manner. Impaired insulin secretion has been reported in GPR39 knockout mice [45,137]. Reduced insulin levels were reported in connection with glucose tolerance tests in 11–12-week-old GPR39^−/−^ mice, as well as a correspondingly lower expression level of the transcription factors insulin promoting factor 1 (IPF1) and hepatocyte nuclear factor 1 homeobox A (HNF-1α) [45]. Similarly, when GPR39^−/−^ mice were fed a HFD, elevated blood glucose levels were observed [138] and when a sucrose-rich diet was fed to 1-year-old GPR39^−/−^ mice, reduced insulin levels were recorded, suggested to result from reduced insulin receptor substrate 2 (IRS-2) levels in the β-cells. No difference was noted in younger mice [137]. Interestingly, GPR39 overexpression in pancreatic β-cells was shown to protect mice from hyperglycemia [139]. Insulin release was indicated to be regulated by GPR39 via Zn^2+^-dependent Ca^2+^ response, which suggests GPR39 activation as a novel means to treat diabetes [140]. However, one study reported that a GPR39 agonist (compound 7 in [75]) stimulated insulin secretion from murine islets isolated from both WT as well as GPR39^−/−^ mice, whereas another GPR39 agonist, TM-N1324, had no effect. These results suggest that the GPR39-dependent regulation of β-cells requires further examination [75]. Recently, GPR39 expression was reported in mouse intestinal enteroendocrine L- and K-cells and was shown to co-localize with insulinotropic polypeptide (GIP) in K-cells [141]. Decreased glucose levels and increased glucose-induced insulin and GIP release were reported in diabetic mice when orally administered with Zn^2+^. Zn^2+^ did not have similar effects in GIP knockout mice [141]. Thus, the effects were verified to be Zn^2+^ and GIP-dependent, but the active role of GPR39 in mediating the Zn^2+^-induced effects needs to be verified.

GPR39 is expressed abundantly in the liver [17], but its functions there remain unknown. Zinc deficiency is often observed in chronic liver disease patients [142], and zinc supplementation is suggested to improve their liver function [143]. One study suggested that a GPR39 agonist, TC-G 1008, ameliorates concanavalin A-induced liver disease, reducing hepatic necrosis [62], and another reported that microRNA-1914 inhibited tumor cell proliferation in a hepatocellular carcinoma (HCC) model by inhibiting GPR39 expression and consequent PI3K/AKT/mTOR activation [144]. Increased expression levels of GPR39 were found in HCC tissues and GPR39 was identified to be a target gene of microRNA-1914, which negatively regulates GPR39 expression in HCC and potentially also in other tissues and conditions [144]. This indicates that whereas GPR39 activation seems beneficial for health in physiological conditions, in pathological conditions, GPR39 inhibition can conversely be a valid treatment strategy.

### 4.5. Gastrointestinal Tract

GPR39 has also been heavily investigated in the GI tract due to obestatin’s suggested role in its activation. GPR39 expression levels in the GI tract and stomach are high [17], and it has been suggested to be an important regulator of the GI but determining its exact functions has been challenging. One study found the gastric emptying of GPR39^−/−^ mice to be nearly twice as fast as of the WT mice. Relevantly, they found increased gastric fluid secretion in the GPR39^−/−^ mice, although no difference was seen in the secretion of gastric acid [133]. More recently, with the help of the novel agonists, GPR39 was identified as an essential regulator of gastric somatostatin secretion [75]. GPR39 activation increased somatostatin secretion and additionally, decreased ghrelin secretion in primary gastric mucosal cells, the response not present when using GPR39^−/−^ cells [75]. GPR39-induced somatostatin secretion could explain the observed increased gastric fluid secretion in GPR39-deficient mice, since somatostatin regulates parietal cell-mediated acid secretion [145]. These results indicate GPR39 to be an important regulator of gastric acid secretion.

Zn^2+^ is frequently used for the treatment of diarrhea and inflammatory bowel disease (IBD) [12,146]. The GPR39-mediated upregulation of NHE likely helps maintain homeostasis during diarrhea, since compromised transporter functions are responsible for the impairment of osmotic gradient and for the loss of Na^+^, Cl^−^ and H_2_O [26,147,148]. The upregulation of NHE-3 increases Na^+^ absorption attenuating diarrhea, but whether this is mediated by GPR39 still requires clarification [148]. GPR39^−/−^ mice suffer from much more severe cholera-toxin-induced diarrhea than WT mice due to increased intestinal fluid secretion, which was shown to result from reduced KCC1-dependent Cl^−^ transport, suggesting a direct role for GPR39 in reducing water loss during diarrhea [94]. Additionally, GPR39 activation was found to increase the expression of clusterin, an anti-apoptotic protein, in colonocytes, enhancing their survival when challenged with the short chain fatty acid butyrate [25]. Altogether, these studies indicate that in addition to Zn^2+^ supplementation, GPR39 activation by agonists may have therapeutic potential in the treatment of diarrhea.

Colonocytes (Caco-2) have been shown to activate GPR39-mediated barrier formation by releasing Zn^2+^ [21]. GPR39 was shown to be involved in increasing the expression of ZO-1 and occluding proteins of the junctional complex and being essential for tight junction formation induced by Zn^2+^ in colonocytes [21]. Correspondingly, the colons of GPR39^−/−^ mice had reduced levels of occludin [149]. This suggested a role for GPR39 activation in IBDs, where the loss of tight junctions is one of the initial signs of the disease [150]. Additionally, recently it was shown that treatment with TC-G 1008 and Zn^2+^ increased tight junction formation and ZO-1 re-organization in T84 intestinal epithelial cells [68]. The effect was shown to be mediated by AMP-activated protein kinase (AMPK), PLC and Ca^2+^/calmodulin-dependent protein kinase kinase β (CaMKKβ), whose inhibitors suppressed the effects [68]. These studies indicate therapeutic potential for GPR39 agonists in the treatment of multiple diseases of the GI tract, where the intestinal barrier function becomes impaired. GPR39-deficient mice were also more prone to develop colitis in a dextran sodium sulfate-induced model than WT mice [149]. IBSs are characterized by repetitive phases of recurrence and remission, and GPR39 expression has been shown to support and speed up the epithelial layer healing during the remission phase: GPR39 upregulated occluding expression, which enhanced the re-formation of tight junctions, as well as increased the proliferation and intestinal crypt formation [149]. These studies suggest that Zn^2+^ supplementation and GPR39 activation may be of therapeutic value during the remission phases of IBD. Not enough data are yet available to determine the effects during the relapses. Zn^2+^ supplementation has been shown to tighten intestinal hyperpermeability, “leaky gut”, a condition present in many gastrointestinal diseases, such as Chron’s disease, where bacteria and endotoxins, such as LPS, are able to enter the circulation and cause systemic low-grade inflammation [151]. These studies suggest that GPR39 signaling is essential for tight junction formation in the intestine, which indicates that the Zn^2+^-induced effects on hyperpermeability are likely GPR39-mediated and GPR39 agonists could be used for the treatment of leaky gut. Additionally, similar mechanisms might take place in the skin, where Zn^2+^ deficiency attenuated GPR39 signaling leads to a loss of tight junctions and could enable pathogen entry through the compromised epithelial barrier. Based on these studies, it seems that GPR39 contributes to the normal functions of the GI tract and might be a novel therapeutic target in the treatment of its disorders, but further research is needed to verify this.

### 4.6. Bone and Cartilage

Bones contain a high amount of Zn^2+^, which is essential for their growth and maintenance [152]. GPR39^−/−^ mice were shown to have weaker bones due to a high mineral-to-matrix ratio and an increased number of active osteoblasts, responsible for bone formation and remodeling [46]. Consistently, GPR39^−/−^ osteoblast cell cultures had reduced collagen and increased mineral contents, suggesting disorganized matrix deposition. Collagen synthesis as well as deposition were shown to be disturbed by the GPR39^−/−^ osteoblasts, and the zinc transporter Zrt- and Irt-like protein 13 (ZIP13) and disintegrin and metalloproteinase with thrombospondin motifs (ADAMTS) 2, 3 and 14, catalyzing collagen cleavage, downregulated [46]. GPR39 was also shown to be involved in osteoblast differentiation and GPR39 expression to be induced by osteoblast differentiation media in the osteoblast cell line MC3T3-E1 [63]. The activation of GPR39 with TC-G 1008 upregulated alkaline phosphatase, osteocalcin and type I collagen expression and increased calcium deposition, indicating GPR39 to be a promotor of differentiation in osteoblasts [63].

GPR39 was shown to become downregulated in connection with osteoarthritis by advanced glycation end products (AGEs) in SW1353 chondrocytes [31]. Treatment with TC-G 1008 ameliorated AGE-induced articular ECM degradation; type II collagen degradation by a downregulation of MMP3 and MMP13 and a promotion of tissue inhibitor metalloproteinases (TIMPs) 1 and 2, and aggregan degradation by a downregulation of ADAMTS 4 and 5. These GPR39-induced effects were suggested to be mediated by the MAPK/nuclear factor-κB (NF-κB) pathway and indicate therapeutic potential for GPR39 agonism in osteoarthritis [31]. Additionally, IL-1β-induced chondrocyte senescence is indicated in the pathogenesis of osteoarthritis, and treatment with TC-G 1008 was reported to ameliorate it [72]. GPR39 was shown to be moderately expressed in human chondrocytes and to be downregulated by IL-1β. TC-G 1008 alleviated IL-1β-induced cell cycle arrest by suppressing the expression of p53, p21 and plasminogen activator inhibitor 1 (PAI-1) and the restoration of SIRT1 expression. A deficiency of SIRT1 eliminated the effect of TC-G 1008 on the cell cycle arrest, indicating that SIRT1 mediated the GPR39-dependent effects [72]. This study brings novel insight into GPR39 signaling affecting the cell cycle, whose disturbance is in a key role in many diseases, such as cancers, viral infections and Alzheimer’s disease [153]. GPR39 was also studied in rheumatoid arthritis, where in addition to downregulating MMP1, MMP3, and MMP13, it was shown to play an active role in reducing inflammation [30]. The treatment of human fibroblast-like synoviocytes with TC-G 1008 ameliorated rheumatoid arthritis markers’ oxidative stress and mitochondrial dysfunction, but also, downregulated the expression of proinflammatory cytokines IL-1β, IL-6 and monocyte chemoattractant protein 1. The effects were suggested to be mediated by Janus kinase (JNK), activating protein 1 (AP-1) and NF-κB pathways [30]. These studies open new possibilities for the use of GPR39 agonists in the treatment of arthritis and bone diseases, such as osteoporosis, where the activation of osteoblasts could help increase bone density.

### 4.7. Cardiovascular Functions

Recently, several studies connecting GPR39 to protection against cardiovascular diseases have emerged. Zn^2+^-induced GPR39 activation was shown to promote vascular cell survival via an activation of cAMP and AKT and an upregulation of platelet-derived growth factor receptor (PDGF-R) and vascular endothelial growth factor A (VEGF-A) [49]. Improvements were also seen in cell adhesion and mobility, endothelial tubule formation and cytoskeletal reorganization, and anti-inflammatory genes, such as heme oxygenase-1 and IL-10 were upregulated. Knockdown of GPR39 in endothelial cells abolished these effects [49]. Two studies have reported GPR39 in vascular calcification. Primary human aortic vascular smooth muscle cells were used to show that ZnSO_4_ reduced phosphate-induced calcification and NF-κB activation via GPR39-induced upregulation of TNF-α induced protein 3 (TNFAIP3), a suppressor of the NF-κB pathway [50]. The silencing of GPR39 or TNFAIP3 eliminated the protection against calcification. ZnSO_4_ also ameliorated vascular calcification in mouse models of chronic renal failure and cholecalciferol overload. An inverse correlation of serum zinc levels and serum calcification propensity was seen in patients with chronic kidney disease [50]. Another study used human valve interstitial cells to demonstrate that Zn^2+^-induced GPR39 activation inhibited apoptosis and osteogenic differentiation via ERK1/2, attenuating calcification and therefore indicating protection against calcific aortic valve disease. Serum Zn^2+^ and GPR39 expression levels were reduced in calcified human aortic valve patients, but treatment with 20 µM Zn^2+^ prevented the reduction, and ZIP13 and ZIP14 deficiencies inhibited the calcification [44]. Testing the potential of GPR39 agonists to inhibit calcification was not studied here but would be of importance. Another study concerning atherosclerosis showed that when aortic endothelial cells were treated with oxidized low-density lipoprotein (ox-LDL), TC-G 1008-induced GPR39 activation reduced oxidative stress and the expression of pro-inflammatory cytokines, chemokines and cellular adhesion molecules, which trigger the attachment of monocytes to endothelial cells. These effects were shown to be mediated by the NF-κB pathway [32]. These studies bring novel insight into the role of GPR39 in the cardiovascular system and notably, indicate further possibilities to investigate GPR39 as a therapeutic target in heart and vascular diseases [32,44,49,50]. Importantly, the latter study further indicates the connection of GPR39 signaling to inflammation and to the NF-κB pathway activation, as also observed in connection with osteoarthritis and rheumatoid arthritis [30,31,32]. Controversially, one study reported NF-κB pathway inhibition by GPR39 [50]. This was observed in vitro under high-phosphate conditions and induced by ZnSO_4_, whereas the other studies used TC-G 1008 [30,31,32]. This could potentially manifest the differences between TC-G 1008 and Zn^2+^-induced GPR39 activation, even more so since NF-κB pathway activation has not been reported in any previous studies with Zn^2+^-induced GPR39 activation, but the non-physiological high-phosphate conditions might also play a role. Further studies are needed to clarify whether TC-G 1008 activates additional pathways via GPR39 compared to Zn^2+^. The first study focusing exclusively on GPR39 in inflammation was published recently. GPR39 expression was found to be upregulated in thioglycollate-induced peritoneal macrophages, and following LPS stimulation, TC-G 1008 increased their IL-10 production in vitro [71]. In a murine model of LPS-induced sepsis, the oral administration of TC-G 1008 increased serum IL-10 levels and survival rates. GPR39 deficiency reduced IL-10 production in both in vivo and in vitro models. The study concludes that GPR39 increased IL-10 production from macrophages in inflammatory conditions and thus, had anti-inflammatory properties [71].

### 4.8. Other Recent Advances in Unraveling the Physiological Functions of GPR39

Several more physiological functions have been quite recently suggested for GPR39, many revealed with the help of the novel GPR39 agonists. Zn^2+^ has been indicated to have a role in salivary secretion disorders and taste [154] and is found in the secretory granules of the salivary glands [155]. GPR39 signaling was shown to take place in ductal salivary gland cells (HSY) [24,27] and later in human submandibular glands and gland cells (HSGs) [156]. Recently, a ZnCl_2_ solution was shown to trigger saliva secretion in humans and using human submandibular gland cells, Zn^2+^ was shown to induce the intracellular Ca^2+^ response and aquaporin-5 translocation to the plasma membrane [156], both in a key role in salivary secretion regulation [157,158]. The effects were shown to be GPR39-mediated by their drastic reduction in GPR39 knockdown cells [156]. GPR39 expression has also been reported in the sperm tail and the acrosome [159,160] and Zn^2+^-induced GPR39 activation was shown to stimulate sperm acrosomal exocytosis [160] and hyper-activated motility [159]. This involved, e.g., GPR39-induced trans-membrane-adenylyl-cyclase and NHE-mediated Ca^2+^ channel activation. These results indicate a possible role for GPR39 in male fertility [161]. A role for GPR39 in myogenic progression has been indicated by one study, where GPR39 induced myoblast differentiation and fast fiber formation [43]. In C2C12 myoblast cells, GPR39 was suggested to be a direct target gene of Zac1, triggering differentiation and fast fiber formation in myoblasts through the activation of CaMK-II, β-catenin inhibition and ERK1/2 dephosphorylation. Zac1/GPR39 deficiency inhibited this response and myoblast differentiation [43]. Interestingly, one study indicated GPR39 activation in hepatitis B virus proliferation. GPR39, activated by TC-G 1008, was shown to induce the transcription of viral promoters and proviral heat shock proteins, suggesting that GPR39 inhibition could be studied as a novel strategy to combat viral replication in cells [66].

Intriguingly, recently GPR39 agonists were studied for the treatment of alcohol use disorder [61]. *GPR39* was found to be hypermethylated and downregulated after chronic alcohol use in non-human primates, rhesus macaques. An acute dose of TC-G 1008 reduced the ethanol preference and ethanol intake of mice drastically, and repeated doses lowered their ethanol escalation in an intermittent ethanol access model. TC-G 1008 increased excitatory neutrotransmission and *Gpr39* and *Bdnf* expression in the nucleus accumbens core, heavily affected by ethanol intake [61]. These results suggest that further studies about TC-G 1008 in addiction disorders would be of importance and indicate GPR39 agonism to be a plausible therapeutic approach for the treatment of alcohol use disorder.

Various zinc transporters have been reported to be overexpressed in different cancers [162,163], but knowledge about the connection between cancer and GPR39 has been clarified only recently. For the past decade, GPR39 has been suggested to have a tumor promoting role in cancer, since its activation increases cell survival, proliferation and migration. Additionally, GPR39 overexpression as well as the activation of the PI3K/AKT and MAPK signaling pathways have been observed in human breast cancer biopsies as well as breast and prostate cancer cells and esophageal and oral squamous cell carcinomas [27,36,164,165,166]. A recent study demonstrated that high GPR39 expression correlated with poor survival in oral squamous cell carcinoma patients. GPR39 via Rho was indicated to regulate YAP, a transcriptional coactivator associated with malignant progression in many cancers, and importantly, the inhibition of GPR39 inhibited growth of the carcinoma [165]. These results indicate that GPR39 may be found to play a role in many additional cancers and could be potentially used as a therapeutic target in their treatment or as a biomarker for poor outcomes.

Furthermore, in a screening for disease associations using sequence kernel association tests, GPR39 sequence variants were indicated to have roles in diseases of hair and their follicles, psoriatic arthropathy, benign essential hypertension, benign hyperplasia of the prostate and nerve function [167], and GPR39 was identified as a biologically relevant candidate gene for pediatric obesity in a study of rare copy number variations [168]. These conditions could be the next ones to be added to the growing repertoire of physiological functions shown to be contributed by GPR39.

## 5. Conclusions and Future Perspectives

The identification of Zn^2+^ as an endogenous ligand for GPR39 has been in a key role in revealing its physiological functions, and the synthetic agonists have further continued to widen our understanding of the molecular mechanisms behind it. Yet, GPR39 is still recurrently included in studies focusing on obestatin [169], and up till now some of these studies suggest that the observed effects of obestatin were indeed GPR39 mediated [170,171,172,173]. Moreover, the question of another ligand existing for GPR39 is still an open one.

Recent findings have indicated the role of GPR39 in inflammation and cardiovascular diseases, which opens up two wide new study areas within GPR39 research. Even though GPR39 is expressed abundantly in the kidney, no study to date has focused on its role there. Additionally, the liver, a central metabolic organ with relatively high GPR39 expression, remains heavily understudied. Zn^2+^ supplementation is a widely used, but not ideal treatment strategy, since Zn^2+^ plays various roles in multiple pathways. The novel agonists have shown great promise and are likely to be of therapeutic potential in a majority of the conditions where GPR39 signaling has been indicated. In the upcoming years, studies utilizing these novel GPR39 agonists will certainly continue to unravel new insights into the physiological functions of GPR39 and essentially, into the therapeutic potential of GPR39 activation. Development of the biased agonists is a novel interesting branch of GPR39 research and could further improve the specificity of treatments. Another interesting branch of research would be the development of GPR39 antagonists, which could be utilized in the treatment of various cancers. Thus, GPR39 remains an important and intriguing research topic, whose physiological functions necessitate further research in order to achieve effective and targeted therapies against the multitude of diseases where Zn^2+^ deficiency plays a role.

## References

[1] Schwartz T.W., Frimurer T.M., Holst B., Rosenkilde M.M., Elling C.E. (2006). Molecular mechanism of 7TM receptor activation—A global toggle switch model. Annu. Rev. Pharmacol. Toxicol..

[2] McKee K.K., Tan C.P., Palyha O.C., Liu J., Feighner S.D., Hreniuk D.L., Smith R.G., Howard A.D., der Ploeg L.H. (1997). Cloning and characterization of two human G protein-coupled receptor genes (GPR38 and GPR39) related to the growth hormone secretagogue and neurotensin receptors. Genomics.

[3] Kaiya H., Kangawa K., Miyazato M. (2013). Molecular evolution of GPCRs: Ghrelin/ghrelin receptors. J. Mol. Endocrinol..

[4] Lauwers E., Landuyt B., Arckens L., Schoofs L., Luyten W. (2006). Obestatin does not activate orphan G protein-coupled receptor GPR39. Biochem. Biophys. Res. Commun..

[5] Hershfinkel M., Moran A., Grossman N., Sekler I. (2001). A zinc-sensing receptor triggers the release of intracellular Ca^2+^ and regulates ion transport. Proc. Natl. Acad. Sci. USA.

[6] Holst B., Egerod K.L., Schild E., Vickers S.P., Cheetham S., Gerlach L.-O., Storjohann L., Stidsen C.E., Jones R., Beck-Sickinger A.G. (2007). GPR39 signaling is stimulated by zinc ions but not by obestatin. Endocrinology.

[7] Yasuda S., Miyazaki T., Munechika K., Yamashita M., Ikeda Y., Kamizono A. (2007). Isolation of Zn^2+^ as an endogenous agonist of GPR39 from fetal bovine serum. J. Recept. Signal Transduct. Res..

[8] Holst B., Holliday N.D., Bach A., Elling C.E., Cox H.M., Schwartz T.W. (2004). Common structural basis for constitutive activity of the ghrelin receptor family. J. Biol. Chem..

[9] Chasapis C.T., Ntoupa P.S.A., Spiliopoulou C.A., Stefanidou M.E. (2020). Recent aspects of the effects of zinc on human health. Arch. Toxicol..

[10] Maret W. (2013). Zinc biochemistry: From a single zinc enzyme to a key element of life. Adv. Nutr..

[11] Roohani N., Hurrell R., Kelishadi R., Schulin R. (2013). Zinc and its importance for human health: An integrative review. J. Res. Med. Sci..

[12] Prasad A.S. (2013). Discovery of human zinc deficiency: Its impact on human health and disease. Adv. Nutr..

[13] Levaot N., Hershfinkel M. (2018). How cellular Zn^2+^ signaling drives physiological functions. Cell Calcium.

[14] Sharir H., Zinger A., Nevo A., Sekler I., Hershfinkel M. (2010). Zinc released from injured cells is acting via the Zn^2+^-sensing receptor, ZnR, to trigger signaling leading to epithelial repair. J. Biol. Chem..

[15] Qian W.J., Gee K.R., Kennedy R.T. (2003). Imaging of Zn^2+^ release from pancreatic β-cells at the level of single exocytotic events. Anal. Chem..

[16] Paoletti P., Vergnano A.M., Barbour B., Casado M. (2009). Zinc at glutamatergic synapses. Neuroscience.

[17] Egerod K.L., Holst B., Petersen P.S., Hansen J.B., Mulder J., Hökfelt T., Schwartz T.W. (2007). GPR39 splice variants versus antisense gene LYPD1: Expression and regulation in gastrointestinal tract, endocrine pancreas, liver, and white adipose tissue. Mol. Endocrinol..

[18] Yasuda S., Ishida J. (2014). GPR39-1b, the 5-transmembrane isoform of GPR39 interacts with neurotensin receptor NTSR1 and modifies its function. J. Recept. Signal Transduct. Res..

[19] Syrovatkina V., Alegre K.O., Dey R., Huang X.Y. (2016). Regulation, Signaling, and Physiological Functions of G-Proteins. J. Mol. Biol..

[20] Dittmer S., Sahin M., Pantlen A., Saxena A., Toutzaris D., Pina A.-L., Geerts A., Golz S., Methner A. (2008). The constitutively active orphan G-protein-coupled receptor GPR39 protects from cell death by increasing secretion of pigment epithelium-derived growth factor. J. Biol. Chem..

[21] Cohen L., Sekler I., Hershfinkel M. (2014). The zinc sensing receptor, ZnR/GPR39, controls proliferation and differentiation of colonocytes and thereby tight junction formation in the colon. Cell Death Dis..

[22] Dong X., Tang S., Zhang W., Gao W., Chen Y. (2016). GPR39 activates proliferation and differentiation of porcine intramuscular preadipocytes through targeting the PI3K/AKT cell signaling pathway. J. Recept. Signal Transduct. Res..

[23] Zhang J.V., Ren P.-G., Avsian-Kretchmer O., Luo C.-W., Rauch R., Klein C., Hsueh A.J.W. (2005). Obestatin, a peptide encoded by the ghrelin gene, opposes ghrelin’s effects on food intake. Science.

[24] Sharir H., Hershfinkel M. (2005). The extracellular zinc-sensing receptor mediates intercellular communication by inducing ATP release. Biochem. Biophys. Res. Commun..

[25] Cohen L., Azriel-Tamir H., Arotsker N., Sekler I., Hershfinkel M. (2012). Zinc sensing receptor signaling, mediated by GPR39, reduces butyrate-induced cell death in HT29 colonocytes via upregulation of clusterin. PLoS ONE.

[26] Azriel-Tamir H., Sharir H., Schwartz B., Herskfinkel M. (2004). Extracellular zinc triggers ERK-dependent activation of Na^+^/H^+^ exchange in colonocytes mediated by the zinc-sensing receptor. J. Biol. Chem..

[27] Asraf H., Salomon S., Nevo A., Sekler I., Mayer D., Hershfinkel M. (2014). The ZnR/GPR39 interacts with the CaSR to enhance signaling in prostate and salivary epithelia. J. Cell. Physiol..

[28] Cho D., Mier J.W., Atkins M.B. (2009). PI3K/Akt/mTOR pathway: A growth and proliferation pathway. Renal Cell Carcinoma: Molecular Targets and Clinical Applications.

[29] Lu Z., Xu S. (2006). ERK1/2 MAP kinases in cell survival and apoptosis. IUBMB Life.

[30] Jing W., Sun W., Zhang N., Zhao C., Yan X. (2019). The protective effects of the GPR39 agonist TC-G 1008 against TNF-α-induced inflammation in human fibroblast-like synoviocytes (FLSs). Eur. J. Pharmacol..

[31] Shan W., Qi J., Li C., Nie X. (2019). Agonism of GPR39 displays protective effects against advanced glycation end-product (AGE)-induced degradation of extracellular matrix in human SW1353 cells. Arch. Biochem. Biophys..

[32] Xu Y., Wang M., Xie Y., Jiang Y., Liu M., Yu S., Wang B., Liu Q. (2019). Activation of GPR39 with the agonist TC-G 1008 ameliorates ox-LDL-induced attachment of monocytes to endothelial cells. Eur. J. Pharmacol..

[33] Holst B., Cygankiewicz A., Jensen T.H., Ankersen M., Schwartz T.W. (2003). High Constitutive Signaling of the Ghrelin Receptor—Identification of a Potent Inverse Agonist. Mol. Endocrinol..

[34] Zhang L., Song J., Zang Z., Tang H., Li W., Lai S., Deng C. (2020). Adaptive evolution of GPR39 in diverse directions in vertebrates. Gen. Comp. Endocrinol..

[35] Shimizu Y., Koyama R., Kawamoto T. (2017). Rho kinase-dependent desensitization of GPR39; a unique mechanism of GPCR downregulation. Biochem. Pharmacol..

[36] Dubi N., Gheber L., Fishman D., Sekler I., Hershfinkel M. (2008). Extracellular zinc and zinc-citrate, acting through a putative zinc-sensing receptor, regulate growth and survival of prostate cancer cells. Carcinogenesis.

[37] Rajagopal S., Shenoy S.K. (2018). GPCR desensitization: Acute and prolonged phases. Cell. Signal..

[38] Holliday N.D., Holst B., Rodionova E.A., Schwartz T.W., Cox H.M. (2007). Importance of constitutive activity and arrestin-independent mechanisms for intracellular trafficking of the ghrelin receptor. Mol. Endocrinol..

[39] González-Maeso J. (2011). GPCR oligomers in pharmacology and signaling. Mol. Brain.

[40] Tena-Campos M., Ramon E., Borroto-Escuela D.O., Fuxe K., Garriga P. (2015). The zinc binding receptor GPR39 interacts with 5-HT1A and GalR1 to form dynamic heteroreceptor complexes with signaling diversity. Biochim. Biophys. Acta.

[41] Zeng F., Wind N., McClenaghan C., Verkuyl J.M., Watson R.P., Nash M.S. (2012). GPR39 is coupled to TMEM16A in intestinal fibroblast-like cells. PLoS ONE.

[42] Kovacs Z., Schacht T., Herrmann A.-K., Albrecht P., Lefkimmiatis K., Methner A. (2014). Protein kinase inhibitor β enhances the constitutive activity of G-protein-coupled zinc receptor GPR39. Biochem. J..

[43] Yang Q., Li Y., Zhang X., Chen D. (2018). Zac1/GPR39 phosphorylating CaMK-II contributes to the distinct roles of Pax3 and Pax7 in myogenic progression. Biochim. Biophys. Acta. Mol. Basis Dis..

[44] Chen Z., Gordillo-Martinez F., Jiang L., He P., Hong W., Wei X., Staines K.A., Macrae V.E., Zhang C., Yu D. (2020). Zinc ameliorates human aortic valve calcification through GPR39 mediated ERK1/2 signaling pathway. Cardiovasc. Res..

[45] Holst B., Egerod K.L., Jin C., Petersen P.S., Østergaard M.V., Hald J., Sprinkel A.M.E., Størling J., Mandrup-Poulsen T., Holst J.J. (2009). G protein-coupled receptor 39 deficiency is associated with pancreatic islet dysfunction. Endocrinology.

[46] Jovanovic M., Schmidt F.N., Guterman-Ram G., Khayyeri H., Hiram-Bab S., Orenbuch A., Katchkovsky S., Aflalo A., Isaksson H., Busse B. (2018). Perturbed bone composition and integrity with disorganized osteoblast function in zinc receptor/Gpr39-deficient mice. FASEB J. Off. Publ. Fed. Am. Soc. Exp. Biol..

[47] Chorin E., Vinograd O., Fleidervish I., Gilad D., Herrmann S., Sekler I., Aizenman E., Hershfinkel M. (2011). Upregulation of KCC2 activity by zinc-mediated neurotransmission via the mZnR/GPR39 receptor. J. Neurosci. Off. J. Soc. Neurosci..

[48] Nishida K., Hasegawa A., Yamasaki S., Uchida R., Ohashi W., Kurashima Y., Kunisawa J., Kimura S., Iwanaga T., Watarai H. (2019). Mast cells play role in wound healing through the ZnT2/GPR39/IL-6 axis. Sci. Rep..

[49] Zhu D., Su Y., Zheng Y., Fu B., Tang L., Qin Y.-X. (2018). Zinc regulates vascular endothelial cell activity through zinc-sensing receptor ZnR/GPR39. Am. J. Physiol. Cell Physiol..

[50] Voelkl J., Tuffaha R., Luong T.T.D., Zickler D., Masyout J., Feger M., Verheyen N., Blaschke F., Kuro-o M., Tomaschitz A. (2018). Zinc Inhibits Phosphate-Induced Vascular Calcification through TNFAIP3-Mediated Suppression of NF-κB. J. Am. Soc. Nephrol. JASN.

[51] Giblin L.J., Chang C.J., Bentley A.F., Frederickson C., Lippard S.J., Frederickson C.J. (2006). Zinc-secreting Paneth cells studied by ZP fluorescence. J. Histochem. Cytochem..

[52] Ishii K., Akita M., Sato M., Tomita H. (1999). Localization of zinc in the rat submandibular gland and the effect of its deficiency on salivary secretion. Ann. Otol. Rhinol. Laryngol..

[53] Perez-Rosello T., Anderson C.T., Schopfer F.J., Zhao Y., Gilad D., Salvatore S.R., Freeman B.A., Hershfinkel M., Aizenman E., Tzounopoulos T. (2013). Synaptic Zn^2+^ inhibits neurotransmitter release by promoting endocannabinoid synthesis. J. Neurosci. Off. J. Soc. Neurosci..

[54] Storjohann L., Holst B., Schwartz T.W. (2008). Molecular mechanism of Zn^2+^ agonism in the extracellular domain of GPR39. FEBS Lett..

[55] Storjohann L., Holst B., Schwartz T.W. (2008). A second disulfide bridge from the N-terminal domain to extracellular loop 2 dampens receptor activity in GPR39. Biochemistry.

[56] Cohen L., Asraf H., Sekler I., Hershfinkel M. (2012). Extracellular pH regulates zinc signaling via an Asp residue of the zinc-sensing receptor (ZnR/GPR39). J. Biol. Chem..

[57] Ganay T., Asraf H., Aizenman E., Bogdanovic M., Sekler I., Hershfinkel M. (2015). Regulation of neuronal pH by the metabotropic Zn(^2+^)-sensing Gq-coupled receptor, mZnR/GPR39. J. Neurochem..

[58] Sakon J., Irwin D., Wilson D.B., Andrew Karplus P. (1997). Structure and mechanism of endo/exocellulase E4 from Thermomonospora fusca. Nat. Struct. Biol..

[59] Boehm M., Hepworth D., Loria P.M., Norquay L.D., Filipski K.J., Chin J.E., Cameron K.O., Brenner M., Bonnette P., Cabral S. (2013). Chemical Probe Identification Platform for Orphan GPCRs Using Focused Compound Screening: GPR39 as a Case Example. ACS Med. Chem. Lett..

[60] Peukert S., Hughes R., Nunez J., He G., Yan Z., Jain R., Llamas L., Luchansky S., Carlson A., Liang G. (2014). Discovery of 2-Pyridylpyrimidines as the First Orally Bioavailable GPR39 Agonists. ACS Med. Chem. Lett..

[61] Carlson V.C.C., Ford M.M., Carlson T.L., Lomniczi A., Grant K.A., Ferguson B., Cervera-Juanes R.P. (2019). Modulation of Gpr39, a G-protein coupled receptor associated with alcohol use in non-human primates, curbs ethanol intake in mice. Neuropsychopharmacol. Off. Publ. Am. Coll. Neuropsychopharmacol..

[62] Muneoka S., Goto M., Nishimura T., Enomoto K., Kadoshima-Yamaoka K., Tomimori Y. (2019). G Protein-Coupled Receptor 39 Agonist Improves Concanavalin A-Induced Hepatitis in Mice. Biol. Pharm. Bull..

[63] Chai X., Zhang W., Chang B., Feng X., Song J., Li L., Yu C., Zhao J., Si H. (2019). GPR39 agonist TC-G 1008 promotes osteoblast differentiation and mineralization in MC3T3-E1 cells. Artif. Cells Nanomed. Biotechnol..

[64] Satianrapapong W., Pongkorpsakol P., Muanprasat C. (2020). A G-protein coupled receptor 39 agonist stimulates proliferation of keratinocytes via an ERK-dependent pathway. Biomed. Pharmacother..

[65] Sato S., Huang X.-P., Kroeze W.K., Roth B.L. (2016). Discovery and Characterization of Novel GPR39 Agonists Allosterically Modulated by Zinc. Mol. Pharmacol..

[66] Goto K., Nishitsuji H., Sugiyama M., Nishida N., Mizokami M., Shimotohno K. (2020). Orchestration of Intracellular Circuits by G Protein-Coupled Receptor 39 for Hepatitis B Virus Proliferation. Int. J. Mol. Sci..

[67] Mo F., Tang Y., Du P., Shen Z., Yang J., Cai M., Zhang Y., Li H., Shen H. (2020). GPR39 protects against corticosterone-induced neuronal injury in hippocampal cells through the CREB-BDNF signaling pathway. J. Affect. Disord..

[68] Pongkorpsakol P., Buasakdi C., Chantivas T., Chatsudthipong V., Muanprasat C. (2019). An agonist of a zinc-sensing receptor GPR39 enhances tight junction assembly in intestinal epithelial cells via an AMPK-dependent mechanism. Eur. J. Pharmacol..

[69] Starowicz G., Jarosz M., Frąckiewicz E., Grzechnik N., Ostachowicz B., Nowak G., Mlyniec K. (2019). Long-lasting antidepressant-like activity of the GPR39 zinc receptor agonist TC-G 1008. J. Affect. Disord..

[70] Młyniec K., Starowicz G., Gaweł M., Frąckiewicz E., Nowak G. (2016). Potential antidepressant-like properties of the TC G-1008, a GPR39 (zinc receptor) agonist. J. Affect. Disord..

[71] Muneoka S., Goto M., Kadoshima-Yamaoka K., Kamei R., Terakawa M., Tomimori Y. (2018). G protein-coupled receptor 39 plays an anti-inflammatory role by enhancing IL-10 production from macrophages under inflammatory conditions. Eur. J. Pharmacol..

[72] Lu H., Wang D., Li H., Zhong J., Lin Y., Xu X., Wang B. (2019). GPR39 agonist TC-G 1008 ameliorates IL-1β-induced chondrocyte senescence. Artif. Cells Nanomed. Biotechnol..

[73] Bassilana F., Carlson A., DaSilva J.A., Grosshans B., Vidal S., Beck V., Wilmeringwetter B., Llamas L.A., Showalter T.B., Rigollier P. (2014). Target identification for a Hedgehog pathway inhibitor reveals the receptor GPR39. Nat. Chem. Biol..

[74] Fjellström O., Larsson N., Yasuda S.-I., Tsuchida T., Oguma T., Marley A., Wennberg-Huldt C., Hovdal D., Fukuda H., Yoneyama Y. (2015). Novel Zn^2+^ Modulated GPR39 Receptor Agonists Do Not Drive Acute Insulin Secretion in Rodents. PLoS ONE.

[75] Frimurer T.M., Mende F., Graae A.-S., Engelstoft M.S., Egerod K.L., Nygaard R., Gerlach L.-O., Hansen J.B., Schwartz T.W., Holst B. (2017). Model-Based Discovery of Synthetic Agonists for the Zn^2+^-Sensing G-Protein-Coupled Receptor 39 (GPR39) Reveals Novel Biological Functions. J. Med. Chem..

[76] Grunddal K.V., Diep T.A., Petersen N., Tough I.R., Skov L.J., Liu L., Buijink J.A., Mende F., Jin C., Jepsen S.L. (2021). Selective release of gastrointestinal hormones induced by an orally active GPR39 agonist. Mol. Metab..

[77] Iglesias M.J., Salgado A., Piñeiro R., Rodiño B.K., Otero M.F., Grigorian L., Gallego R., Diéguez C., Gualillo O., González-Juanatey J.R. (2007). Lack of effect of the ghrelin gene-derived peptide obestatin on cardiomyocyte viability and metabolism. J. Endocrinol. Investig..

[78] Yamamoto I., Kimura N., Arai T., Tanaka M. (2009). cDNA cloning and mRNA expression of bovine GPR39. J. Vet. Med. Sci..

[79] Yamamoto I., Numao M., Sakaguchi Y., Tsushima N., Tanaka M. (2007). Molecular characterization of sequence and expression of chicken GPR39. Gen. Comp. Endocrinol..

[80] Yamamoto I., Sakaguchi Y., Numao M., Tsukada A., Tsushima N., Tanaka M. (2007). Primary structure and tissue distribution of GPR39 messenger ribonucleic acid in Japanese quail, Coturnix japonica. Poult. Sci..

[81] Zhang Y., Liu Y., Huang X., Liu X., Jiao B., Meng Z., Zhu P., Li S., Lin H., Cheng C.H.K. (2008). Two alternatively spliced GPR39 transcripts in seabream: Molecular cloning, genomic organization, and regulation of gene expression by metabolic signals. J. Endocrinol..

[82] Jackson V.R., Nothacker H.-P., Civelli O. (2006). GPR39 receptor expression in the mouse brain. Neuroreport.

[83] Metsuyanim S., Harari-Steinberg O., Buzhor E., Omer D., Pode-Shakked N., Ben-Hur H., Halperin R., Schneider D., Dekel B. (2009). Expression of stem cell markers in the human fetal kidney. PLoS ONE.

[84] Catalán V., Gómez-Ambrosi J., Rotellar F., Silva C., Gil M.J., Rodríguez A., Cienfuegos J.A., Salvador J., Frühbeck G. (2007). The obestatin receptor (GPR39) is expressed in human adipose tissue and is down-regulated in obesity-associated type 2 diabetes mellitus. Clin. Endocrinol..

[85] Fontenot E., DeVente J.E., Seidel E.R. (2007). Obestatin and ghrelin in obese and in pregnant women. Peptides.

[86] Aoi W., Marunaka Y. (2014). Importance of pH Homeostasis in Metabolic Health and Diseases: Crucial Role of Membrane Proton Transport. BioMed Res. Int..

[87] Takahashi K.I., Copenhagen D.R. (1996). Modulation of neuronal function by intracellular pH. Neurosci. Res..

[88] Blachier F., de Sá Resende A., da Silva Fogaça Leite G., Vasques da Costa A., Lancha Junior A.H. (2018). Colon epithelial cells luminal environment and physiopathological consequences: Impact of nutrition and exercise. Nutrire.

[89] Hachem J.P., Behne M., Aronchik I., Demerjian M., Feingold K.R., Elias P.M., Mauro T.M. (2005). Extracellular pH controls NHE1 expression in epidermis and keratinocytes: Implications for barrier repair. J. Investig. Dermatol..

[90] Ardura J.A., Friedman P.A. (2011). Regulation of G protein-coupled receptor function by Na^+^/H^+^ exchange regulatory factors. Pharmacol. Rev..

[91] Gillen C.M., Brill S., Payne J.A., Forbush B. (1996). Molecular cloning and functional expression of the K-Cl cotransporter from rabbit, rat, and human: A new member of the cation-chloride cotransporter family. J. Biol. Chem..

[92] Rivera C., Voipio J., Payne J.A., Ruusuvuori E., Lahtinen H., Lamsa K., Pirvola U., Saarma M., Kaila K. (1999). The K^+^/Cl^−^ co-transporter KCC2 renders GABA hyperpolarizing during neuronal maturation. Nature.

[93] Payne J.A., Stevenson T.J., Donaldson L.F. (1996). Molecular characterization of a putative K-Cl cotransporter in rat brain: A neuronal-specific isoform. J. Biol. Chem..

[94] Sunuwar L., Asraf H., Donowitz M., Sekler I., Hershfinkel M. (2017). The Zn^2+^-sensing receptor, ZnR/GPR39, upregulates colonocytic Cl^-^ absorption, via basolateral KCC1, and reduces fluid loss. Biochim. Biophys. Acta. Mol. Basis Dis..

[95] Mero M., Asraf H., Sekler I., Taylor K.M., Hershfinkel M. (2019). ZnR/GPR39 upregulation of K^+^/Cl^−^-cotransporter 3 in tamoxifen resistant breast cancer cells. Cell Calcium.

[96] Chakraborty M., Asraf H., Sekler I., Hershfinkel M. (2021). ZnR/GPR39 controls cell migration by orchestrating recruitment of KCC3 into protrusions, re-organization of actin and activation of MMP. Cell Calcium.

[97] McAllister B.B., Dyck R.H. (2017). Zinc transporter 3 (ZnT3) and vesicular zinc in central nervous system function. Neurosci. Biobehav. Rev..

[98] Cole T.B., Wenzel H.J., Kafer K.E., Schwartzkroin P.A., Palmiter R.D. (1999). Elimination of zinc from synaptic vesicles in the intact mouse brain by disruption of the ZnT3 gene. Proc. Natl. Acad. Sci. USA.

[99] Ketterman J.K., Li Y.V. (2008). Presynaptic evidence for zinc release at the mossy fiber synapse of rat hippocampus. J. Neurosci. Res..

[100] Qian J., Noebels J.L. (2005). Visualization of transmitter release with zinc fluorescence detection at the mouse hippocampal mossy fibre synapse. J. Physiol..

[101] Besser L., Chorin E., Sekler I., Silverman W.F., Atkin S., Russell J.T., Hershfinkel M. (2009). Synaptically released zinc triggers metabotropic signaling via a zinc-sensing receptor in the hippocampus. J. Neurosci. Off. J. Soc. Neurosci..

[102] Adlard P.A., Parncutt J.M., Finkelstein D.I., Bush A.I. (2010). Cognitive loss in zinc transporter-3 knock-out mice: A phenocopy for the synaptic and memory deficits of Alzheimer’s disease?. J. Neurosci..

[103] Suh S.W., Won S.J., Hamby A.M., Yoo B.H., Fan Y., Sheline C.T., Tamano H., Takeda A., Liu J. (2009). Decreased brain zinc availability reduces hippocampal neurogenesis in mice and rats. J. Cereb. Blood Flow Metab..

[104] Cole T.B., Robbins C.A., Wenzel H.J., Schwartzkroin P.A., Palmiter R.D. (2000). Seizures and neuronal damage in mice lacking vesicular zinc. Epilepsy Res..

[105] Wang J., Um P., Dickerman B.A., Liu J. (2018). Zinc, magnesium, selenium and depression: A review of the evidence, potential mechanisms and implications. Nutrients.

[106] Młyniec K., Doboszewska U., Szewczyk B., Sowa-Kućma M., Misztak P., Piekoszewski W., Trela F., Ostachowicz B., Nowak G. (2014). The involvement of the GPR39-Zn(^2+^)-sensing receptor in the pathophysiology of depression. Studies in rodent models and suicide victims. Neuropharmacology.

[107] Młyniec K., Nowak G. (2013). GPR39 up-regulation after selective antidepressants. Neurochem. Int..

[108] Młyniec K., Gaweł M., Librowski T., Reczyński W., Bystrowska B., Holst B. (2015). Investigation of the GPR39 zinc receptor following inhibition of monoaminergic neurotransmission and potentialization of glutamatergic neurotransmission. Brain Res. Bull..

[109] Młyniec K., Nowak G. (2015). Up-regulation of the GPR39 Zn^2+^-sensing receptor and CREB/BDNF/TrkB pathway after chronic but not acute antidepressant treatment in the frontal cortex of zinc-deficient mice. Pharmacol. Rep..

[110] Młyniec K., Budziszewska B., Holst B., Ostachowicz B., Nowak G. (2014). GPR39 (zinc receptor) knockout mice exhibit depression-like behavior and CREB/BDNF down-regulation in the hippocampus. Int. J. Neuropsychopharmacol..

[111] Yang T., Nie Z., Shu H., Kuang Y., Chen X., Cheng J., Yu S., Liu H. (2020). The Role of BDNF on Neural Plasticity in Depression. Front. Cell. Neurosci..

[112] Omar N.N., Tash R.F. (2017). Fluoxetine coupled with zinc in a chronic mild stress model of depression: Providing a reservoir for optimum zinc signaling and neuronal remodeling. Pharmacol. Biochem. Behav..

[113] Borroto-Escuela D.O., Narvaez M., Marcellino D., Parrado C., Narvaez J.A., Tarakanov A.O., Agnati L.F., Díaz-Cabiale Z., Fuxe K. (2010). Galanin receptor-1 modulates 5-hydroxtryptamine-1A signaling via heterodimerization. Biochem. Biophys. Res. Commun..

[114] Jia W., Song Y., Yang L., Kong J., Boczek T., He Z., Wang Y., Zhang X., Hu H., Shao D. (2020). The changes of serum zinc, copper, and selenium levels in epileptic patients: A systematic review and meta-analysis. Expert Rev. Clin. Pharmacol..

[115] Doboszewska U., Młyniec K., Wlaź A., Poleszak E., Nowak G., Wlaź P. (2019). Zinc signaling and epilepsy. Pharmacol. Ther..

[116] Heydarian F., Nakhaei A.A., Majd H.M., Bakhtiari E. (2020). Zinc deficiency and febrile seizure: A systematic review and meta-analysis. Turk. J. Pediatr..

[117] Arul J., Kommu P.P.K., Kasinathan A., Ray L., Krishnan L. (2020). Zinc Status and Febrile Seizures: Results from a Cross-sectional Study. J. Neurosci. Rural Pract..

[118] Woo N.S., Lu J., England R., McClellan R., Dufour S., Mount D.B., Deutch A.Y., Lovinger D.M., Delpire E. (2002). Hyperexcitability and epilepsy associated with disruption of the mouse neuronal-specific K-Cl cotransporter gene. Hippocampus.

[119] Zhu L., Lovinger D., Delpire E. (2005). Cortical neurons lacking KCC2 expression show impaired regulation of intracellular chloride. J. Neurophysiol..

[120] Gilad D., Shorer S., Ketzef M., Friedman A., Sekler I., Aizenman E., Hershfinkel M. (2015). Homeostatic regulation of KCC2 activity by the zinc receptor mZnR/GPR39 during seizures. Neurobiol. Dis..

[121] Chen N.-N., Zhao D.-J., Sun Y.-X., Wang D.-D., Ni H. (2019). Long-Term Effects of Zinc Deficiency and Zinc Supplementation on Developmental Seizure-Induced Brain Damage and the Underlying GPR39/ZnT-3 and MBP Expression in the Hippocampus. Front. Neurosci..

[122] Sunuwar L., Gilad D., Hershfinkel M. (2017). The zinc sensing receptor, ZnR/GPR39, in health and disease. Front. Biosci..

[123] Xu Y., Xiao G., Liu L., Lang M. (2019). Zinc transporters in Alzheimer’s disease. Mol. Brain.

[124] Huang X., Atwood C.S., Moir R.D., Hartshorn M.A., Vonsattel J.P., Tanzi R.E., Bush A.I. (1997). Zinc-induced Alzheimer’s Aβ1-40 aggregation is mediated by conformational factors. J. Biol. Chem..

[125] Takeda A., Tamano H., Tempaku M., Sasaki M., Uematsu C., Sato S., Kanazawa H., Datki Z.L., Adlard P.A., Bush A.I. (2017). Extracellular Zn^2+^ is essential for amyloid β1-42-induced cognitive decline in the normal brain and its rescue. J. Neurosci..

[126] Abramovitch-Dahan C., Asraf H., Bogdanovic M., Sekler I., Bush A.I., Hershfinkel M. (2016). Amyloid β attenuates metabotropic zinc sensing receptor, mZnR/GPR39, dependent Ca^2+^, ERK1/2 and Clusterin signaling in neurons. J. Neurochem..

[127] Loef M., Schrauzer G.N., Walach H. (2011). Selenium and alzheimer’s disease: A systematic review. J. Alzheimer’s Dis..

[128] Farbood Y., Sarkaki A., Mahdavinia M., Ghadiri A., Teimoori A., Seif F., Dehghani M.A., Navabi S.P. (2020). Protective Effects of Co-administration of Zinc and Selenium Against Streptozotocin-Induced Alzheimer’s Disease: Behavioral, Mitochondrial Oxidative Stress, and GPR39 Expression Alterations in Rats. Neurotox. Res..

[129] Kogan S., Sood A., Garnick M.S. (2017). Zinc and Wound Healing: A Review of Zinc Physiology and Clinical Applications. Wounds Compend. Clin. Res. Pract..

[130] Bin B.H., Hojyo S., Seo J., Hara T., Takagishi T., Mishima K., Fukada T. (2018). The role of the slc39a family of zinc transporters in zinc homeostasis in skin. Nutrients.

[131] Fukunaka A., Fujitani Y. (2018). Role of zinc homeostasis in the pathogenesis of diabetes and obesity. Int. J. Mol. Sci..

[132] Tremblay F., Perreault M., Klaman L.D., Tobin J.F., Smith E., Gimeno R.E. (2007). Normal food intake and body weight in mice lacking the G protein-coupled receptor GPR39. Endocrinology.

[133] Moechars D., Depoortere I., Moreaux B., de Smet B., Goris I., Hoskens L., Daneels G., Kass S., Ver Donck L., Peeters T. (2006). Altered gastrointestinal and metabolic function in the GPR39-obestatin receptor-knockout mouse. Gastroenterology.

[134] Petersen P.S., Jin C., Madsen A.N., Rasmussen M., Kuhre R., Egerod K.L., Nielsen L.B., Schwartz T.W., Holst B. (2011). Deficiency of the GPR39 receptor is associated with obesity and altered adipocyte metabolism. FASEB J. Off. Publ. Fed. Am. Soc. Exp. Biol..

[135] Pound L.D., Sarkar S.A., Benninger R.K.P., Wang Y., Suwanichkul A., Shadoan M.K., Printz R.L., Oeser J.K., Lee C.E., Piston D.W. (2009). Deletion of the mouse Slc30a8 gene encoding zinc transporter-8 results in impaired insulin secretion. Biochem. J..

[136] Nicolson T.J., Bellomo E.A., Wijesekara N., Loder M.K., Baldwin J.M., Gyulkhandanyan A.V., Koshkin V., Tarasov A.I., Carzaniga R., Kronenberger K. (2009). Insulin storage and glucose homeostasis in mice null for the granule zinc transporter ZnT8 and studies of the type 2 diabetes-associated variants. Diabetes.

[137] Tremblay F., Richard A.-M.T., Will S., Syed J., Stedman N., Perreault M., Gimeno R.E. (2009). Disruption of G protein-coupled receptor 39 impairs insulin secretion in vivo. Endocrinology.

[138] Verhulst P.J., Lintermans A., Janssen S., Loeckx D., Himmelreich U., Buyse J., Tack J., Depoortere I. (2011). GPR39, a receptor of the ghrelin receptor family, plays a role in the regulation of glucose homeostasis in a mouse model of early onset diet-induced obesity. J. Neuroendocrinol..

[139] Egerod K.L., Jin C., Petersen P.S., Wierup N., Sundler F., Holst B., Schwartz T.W. (2011). β-Cell Specific Overexpression of GPR39 Protects against Streptozotocin-Induced Hyperglycemia. Int. J. Endocrinol..

[140] Moran B.M., Abdel-Wahab Y.H.A., Vasu S., Flatt P.R., McKillop A.M. (2016). GPR39 receptors and actions of trace metals on pancreatic beta cell function and glucose homoeostasis. Acta Diabetol..

[141] Moran B.M., Miskelly M.G., Abdel-Wahab Y.H.A., Flatt P.R., McKillop A.M. (2019). Zinc-induced activation of GPR39 regulates glucose homeostasis through glucose-dependent insulinotropic polypeptide secretion from enteroendocrine K-cells. Biol. Chem..

[142] Katayama K. (2020). Zinc and protein metabolism in chronic liver diseases. Nutr. Res..

[143] Mohommad M.K., Zhou Z., Cave M., Barve A., McClain C.J. (2012). Zinc and liver disease. Nutr. Clin. Pract..

[144] Sun L., Wang L., Chen T., Yao B., Wang Y., Li Q., Yang W., Liu Z. (2019). microRNA-1914, which is regulated by lncRNA DUXAP10, inhibits cell proliferation by targeting the GPR39-mediated PI3K/AKT/mTOR pathway in HCC. J. Cell. Mol. Med..

[145] Engevik A.C., Kaji I., Goldenring J.R. (2020). The physiology of the gastric parietal cell. Physiol. Rev..

[146] Vaghari-Tabari M., Jafari-Gharabaghlou D., Sadeghsoltani F., Hassanpour P., Qujeq D., Rashtchizadeh N., Ghorbanihaghjo A. (2020). Zinc and Selenium in Inflammatory Bowel Disease: Trace Elements with Key Roles?. Biol. Trace Elem. Res..

[147] Camilleri M., Sellin J.H., Barrett K.E. (2017). Pathophysiology, Evaluation, and Management of Chronic Watery Diarrhea. Gastroenterology.

[148] Das S., Jayaratne R., Barrett K.E. (2018). The Role of Ion Transporters in the Pathophysiology of Infectious Diarrhea. Cell. Mol. Gastroenterol. Hepatol..

[149] Sunuwar L., Medini M., Cohen L., Sekler I., Hershfinkel M. (2016). The zinc sensing receptor, ZnR/GPR39, triggers metabotropic calcium signalling in colonocytes and regulates occludin recovery in experimental colitis. Philos. Trans. R. Soc. Lond. B Biol. Sci..

[150] Landy J., Ronde E., English N., Clark S.K., Hart A.L., Knight S.C., Ciclitira P.J., Al-Hassi H.O. (2016). Tight junctions in inflammatory bowel diseases and inflammatory bowel disease associated colorectal cancer. World J. Gastroenterol..

[151] Skrovanek S. (2014). Zinc and gastrointestinal disease. World J. Gastrointest. Pathophysiol..

[152] Huang T., Yan G., Guan M. (2020). Zinc homeostasis in bone: Zinc transporters and bone diseases. Int. J. Mol. Sci..

[153] Zhivotovsky B., Orrenius S. (2010). Cell cycle and cell death in disease: Past, present and future. J. Intern. Med..

[154] Tanaka M. (2002). Secretory function of the salivary gland in patients with taste disorders or xerostomia: Correlation with zinc deficiency. Acta Oto-Laryngol..

[155] Frederickson C.J., Perez-Clausell J., Danscher G. (1987). Zinc-containing 7S-NGF complex. Evidence from zinc histochemistry for localization in salivary secretory granules. J. Histochem. Cytochem..

[156] Kim Y.-J., Jo Y., Lee Y.-H., Park K., Park H.-K., Choi S.-Y. (2019). Zn^2+^ stimulates salivary secretions via metabotropic zinc receptor ZnR/GPR39 in human salivary gland cells. Sci. Rep..

[157] Ambudkar I.S. (2016). Calcium signalling in salivary gland physiology and dysfunction. J. Physiol..

[158] Ishikawa Y., Cho G., Yuan Z., Inoue N., Nakae Y. (2006). Aquaporin-5 water channel in lipid rafts of rat parotid glands. Biochim. Biophys. Acta-Biomembr..

[159] Allouche-Fitoussi D., Bakhshi D., Breitbart H. (2019). Signaling pathways involved in human sperm hyperactivated motility stimulated by Zn^2+^. Mol. Reprod. Dev..

[160] Michailov Y., Ickowicz D., Breitbart H. (2014). Zn^2+^-stimulation of sperm capacitation and of the acrosome reaction is mediated by EGFR activation. Dev. Biol..

[161] Allouche-Fitoussi D., Breitbart H. (2020). The Role of Zinc in Male Fertility. Int. J. Mol. Sci..

[162] Bafaro E., Liu Y., Xu Y., Dempski R.E. (2017). The emerging role of zinc transporters in cellular homeostasis and cancer. Signal Transduct. Target. Ther..

[163] Pan Z., Choi S., Ouadid-Ahidouch H., Yang J.M., Beattie J.H., Korichneva I. (2017). Zinc transporters and dysregulated channels in cancers. Front. Biosci. -Landmark.

[164] Xie F., Liu H., Zhu Y.-H., Qin Y.-R., Dai Y., Zeng T., Chen L., Nie C., Tang H., Li Y. (2011). Overexpression of GPR39 contributes to malignant development of human esophageal squamous cell carcinoma. BMC Cancer.

[165] Jiang Y., Li T., Wu Y., Xu H., Xie C., Dong Y., Zhong L., Wang Z., Zhao H., Zhou Y. (2020). GPR39 Overexpression in OSCC Promotes YAP-Sustained Malignant Progression. J. Dent. Res..

[166] Ventura-Bixenshpaner H., Asraf H., Chakraborty M., Elkabets M., Sekler I., Taylor K.M., Hershfinkel M. (2018). Enhanced ZnR/GPR39 Activity in Breast Cancer, an Alternative Trigger of Signaling Leading to Cell Growth. Sci. Rep..

[167] Dershem R., Metpally R.P.R., Jeffreys K., Krishnamurthy S., Smelser D.T., Hershfinkel M., Center R.G., Carey D.J., Robishaw J.D., Breitwieser G.E. (2019). Rare-variant pathogenicity triage and inclusion of synonymous variants improves analysis of disease associations of orphan G protein-coupled receptors. J. Biol. Chem..

[168] Selvanayagam T., Walker S., Gazzellone M.J., Kellam B., Cytrynbaum C., Stavropoulos D.J., Li P., Birken C.S., Hamilton J., Weksberg R. (2018). Genome-wide copy number variation analysis identifies novel candidate loci associated with pediatric obesity. Eur. J. Hum. Genet. EJHG.

[169] Green B.D., Grieve D.J. (2018). Biochemical properties and biological actions of obestatin and its relevence in type 2 diabetes. Peptides.

[170] Bao L.-Z., Shen M., Qudirat H., Shi J.-B., Su T., Song J.-W., Wang Z.-K., Zhao X.-X., Jing Q., Zheng X. (2019). Obestatin ameliorates water retention in chronic heart failure by downregulating renal aquaporin 2 through GPR39, V2R and PPARG signaling. Life Sci..

[171] Sánchez-Temprano A., Relova J.L., Camiña J.P., Pazos Y. (2021). Concurrent Akt, ERK1/2 and AMPK Activation by Obestatin Inhibits Apoptotic Signaling Cascades on Nutrient-Deprived PC12 Cells. Cell. Mol. Neurobiol..

[172] Santos-Zas I., Negroni E., Mamchaoui K., Mosteiro C.S., Gallego R., Butler-Browne G.S., Pazos Y., Mouly V., Camiña J.P. (2017). Obestatin Increases the Regenerative Capacity of Human Myoblasts Transplanted Intramuscularly in an Immunodeficient Mouse Model. Mol. Ther. J. Am. Soc. Gene Ther..

[173] Cid-Díaz T., Santos-Zas I., González-Sánchez J., Gurriarán-Rodríguez U., Mosteiro C.S., Casabiell X., García-Caballero T., Mouly V., Pazos Y., Camiña J.P. (2017). Obestatin controls the ubiquitin-proteasome and autophagy-lysosome systems in glucocorticoid-induced muscle cell atrophy. J. Cachexia. Sarcopenia Muscle.

